# Characterizing camel milk constituents in the Sprague–Dawley rats' blood: A comparative profile with cow's milk attributes

**DOI:** 10.1002/fsn3.3672

**Published:** 2023-09-08

**Authors:** Amal M. Al‐Saffar, Nadia Y. Al‐Qattan, Mona A. Al‐Sughayer

**Affiliations:** ^1^ Department of Biological Sciences, Faculty of Science Kuwait University Safat Kuwait

**Keywords:** camel milk, diabetes, index of atherogenicity, lipoproteins

## Abstract

A comparative study of the hypoglycemic and hypotriglyceridemic effects of raw and pasteurized camel milk was conducted on the lipid profiles of six groups of male normal and diabetic Sprague–Dawley rats (age, 7–8 weeks, 5/group). The standard procedure to induce diabetes in rats was to administer a single intraperitoneal injection of streptozotocin (55 mg/kg, body weight). Rats with fasting blood glucose levels higher than 250 mg/dL were considered diabetics. Raw and pasteurized camel milk reduced blood glucose and triacylglycerol (TAG) levels in diabetic rats. Raw camel milk showed no significant effect on low‐density blood and lipoprotein cholesterol in diabetic rats. Contrarily, pasteurized camel milk significantly increased high‐density lipoprotein cholesterol in diabetic rats. Comparative analysis revealed camel milk with higher levels of lactose, vitamin C, and mono‐unsaturated fatty acids (MUFA) and lower levels of fat, protein, ω6:ω3 PUFA (poly‐unsaturated fatty acids), and index of atherogenicity than cow's milk. Experiments with cow's milk on rats were not carried out because of their characterized inexplicable traits. This novel study suggests that camel milk can be substituted for diabetic patients in place of cow's milk, assuring no side effects besides their effective hypoglycemic and hypotriglyceridemic qualities.

## Graphical abstract

Raw and pasteurized camel milk has hypoglycemic and hypotriglyceridemic properties. Pasteurized camel milk increased high‐density lipoprotein cholesterol in streptozotocin‐induced diabetic rats and decreased blood glucose and triacylglycerol. Fatty acid profiles suggest beneficial properties of consuming pasteurized camel milk and calculated index of atherogenicity recommend camel milk as a better alternative to other milk.

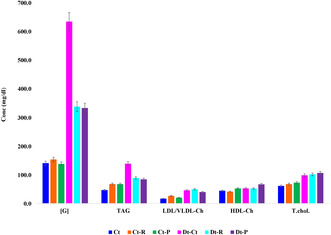

## 1 INTRODUCTION

Diabetes is a major risk factor for atherosclerotic cardiovascular diseases, leading to increased morbidity and mortality (Balakumar et al., 2016; Hudspeth, 2018). Diabetes causes remarkable disturbances in lipid metabolism, including qualitative and quantitative lipid abnormalities, in children and adolescents (Athyros et al., 2018). Hypertriglyceridemia, a feature of untreated diabetes, is characterized by increased low‐density lipoprotein cholesterol (LDL‐Ch) and simultaneously decreased high‐density lipoprotein cholesterol (HDL‐Ch) (Amor et al., 2020; Hirano, 2018; Qaid et al., 2016).

Camel milk is known to have therapeutic effects against many diseases such as dropsy, jaundice, spleen problems, tuberculosis, asthma, anemia, cancer, acute respiratory distress syndrome, and diabetes (Hussain et al., 2021; Singh et al., 2017). Furthermore, it has been shown to improve wound healing in diabetic rats (Abdulrahman et al., 2016; Ebaid et al., 2015; Gader & Al‐Haider, 2016; Galali & Al‐Dmoor, 2019; Mihic et al., 2016; Mirmiran et al., 2017). In some parts of the world, camel milk is commonly fed to children to strengthen their immune systems and improve other biological functions (Abrhaley & Leta, 2018; Gallagher, 2008; Malik et al., 2012; Rasheed, 2017; Zhu et al., 2016). Camel milk lacks β‐*lactoglobulin* (Polidori et al., 2021), but has distinctive amounts of *β*‐casein (Hinz et al., 2012; Karaman et al., 2021; Kula, 2016) and has high vitamin‐C (Christie‐David et al., 2015; Pehlivan, 2017). However, these proteins are considered the major antigens in cow milk and are responsible for hypersensitivity reactions in infants (Park & Haenlein, 2021; Pensabene et al., 2018).

Recently, interest in the therapeutic potential of camel milk for the treatment of diabetes mellitus (DM) has been increasing. Many studies have shown the hypoglycemic effects of camel milk in humans and animals with type‐1 diabetes (Al‐Numair et al., 2011; Ejtahed et al., 2015). An extensive review by Mirmiran et al. (2017) revealed that camel milk reduces plasma glucose and blood glycosylated hemoglobin levels and increases plasma insulin and hemoglobin levels. They also found that diabetic rats fed camel milk showed decreased activities of gluconeogenic enzymes such as glucose 6‐phosphatase and fructose 1,6‐bisphosphatase and increased activities of glucokinase, glucose 6‐phosphate dehydrogenase, and glycogen. Camel milk has also been shown to reduce insulin intake and is considered an effective supplement in the management of type‐1 diabetes (Hussain et al., 2021; Shori, 2015). The hypoglycemic properties can be attributed to the presence of insulin and insulin‐like proteins in camel milk and its ability to remain in an uncoagulated state in an acidic environment (Agrawal et al., 2005; Rasheed, 2017). Ejtahed et al. (2015) confirmed significant hypoglycemic effects in type‐1 diabetes patients who consumed pasteurized camel milk, contrary to their ineffectiveness in type‐2 diabetes patients. However, these studies have focused on the effect of camel milk intake on the blood lipid profile of diabetic subjects. Studies (Agrawal et al., 2005; Al‐Numair, 2010; Al‐Numair et al., 2011) showed that diabetic and non‐diabetic rats fed camel milk showed lower plasma levels of total cholesterol. Furthermore, El‐Zahar et al. (2021) revealed camel milk with an increase in HDL‐Ch and corresponding decrease in total cholesterol levels in diabetic rats. Similar studies showed the varied lipid profile of camel milk and its effect on children (Konuspayeva et al., 2008; Zibaee et al., 2015).

This study aims to investigate the hypoglycemic and hypotriglyceridemic effects of raw and pasteurized camel milk collected from the State of Kuwait. Furthermore, the effects of raw and pasteurized camel milk on the blood lipid profiles (HDL‐Ch and LDL/very low‐density lipoprotein cholesterol (VLDL) cholesterol) of normal and streptozotocin (STZ)‐induced diabetic rats were determined. The correlated factors between the fatty acid pattern of raw and pasteurized camel milk and the blood lipid profile of non‐diabetic and diabetic rats were evaluated. Additionally, the effects of raw and pasteurized cow's milk were compared to validate the preference for consuming camel milk over cow's milk.

## 2 MATERIALS AND METHODS

### 2.1 Animals and diet regimes

Male Sprague–Dawley rats aged 7–8 weeks and weighing 152.5 ± 0.5 g used in this study were housed in polypropylene cages at 23 ± 1°C and 40% relative humidity under a 12 h dark–light regime. The animals were maintained on a normal diet (Special Diets Services) and had ad libitum access to filtered tap water. These test animals were used following the guidelines of the National Research Council (1996) and updates by the Experimental Animals Use and Welfare Committee, Department of Biological Sciences, Kuwait University.

Diabetes was induced with a single intraperitoneal injection of freshly prepared STZ (55 mg/kg body weight) in 0.5 mL of normal saline. Fasting blood sugar was checked for 3 days after the STZ injection. Blood was collected from the tail vein and tested using a blood glucometer (Accu‐Check Performa). Animals with blood glucose levels higher than 250 mg/dL were classified as severely diabetic.

The animals were selected into six groups (*n* = 5/group): group‐1 (Ct), non‐diabetic control rats maintained on a normal rodent diet (RM1) and water; group‐2 (Ct‐R), non‐diabetic rats fed with normal RM1 diet and raw camel milk; group‐3 (Ct‐P), non‐diabetic rats fed with RM1 diet and pasteurized camel milk, group‐4 (Dt‐Ct), diabetic control rats fed with normal RM1 diet and water; group‐5 (Dt‐R), diabetic rats fed with RM1 diet and raw camel milk; and group 6 (Dt‐P), diabetic rats fed with normal RM1 diet and pasteurized camel milk. Throughout the experimental period, all the animals were fed with camel milk (150 mL/day) for 5 weeks instead of water, except group 1 and group 4.

### 2.2 Samples and data collection

The behavioral and visible changes in the physical condition of the animal's weight were regularly monitored. The diabetic rats of the Dt‐Ct group 4 were handled carefully, particularly as they consumed more water and food. During the experiment, their physical condition deteriorated with time and they became frail. The diabetic rats in the Dt‐R and Dt‐P groups were also continuously monitored as they consumed more milk than the Ct‐R and Ct‐P group rats. Blood was drawn on a weekly basis from the tail veins of the animals, and their fasting blood glucose level was monitored. Later, animals were subjected to fasting for 12 h and then anesthetized with diethyl ether. By cardiac puncture, blood was drawn and centrifuged (Eppendorf 5415R, Germany) at 1.2 g/mL density for 20 min at 4°C. The serum samples were collected and dispensed into 50 μL aliquots, and stored at −40°C until analysis. Camel milk purchased from a local supplier was dispensed in two replicates. One replicate was stored raw at 4°C, and the other replicate was pasteurized by heating at 63°C in a water bath (Julabo‐SW22) for 30 min, followed by rapid chilling and storing at 4°C.

### 2.3 Serum analytical studies

Serum glucose and TAG levels were determined using the WAKO Diagnostics L‐Type Triglyceride M assay kit (Wako Chemicals USA, Inc.). Serum HDL‐Ch and LDL/VLDL‐cholesterol levels were measured using the Quantification Colorimetric‐Fluorometric Kit (BioVision).

### 2.4 Milk analytical studies

**FIGURE 1:**
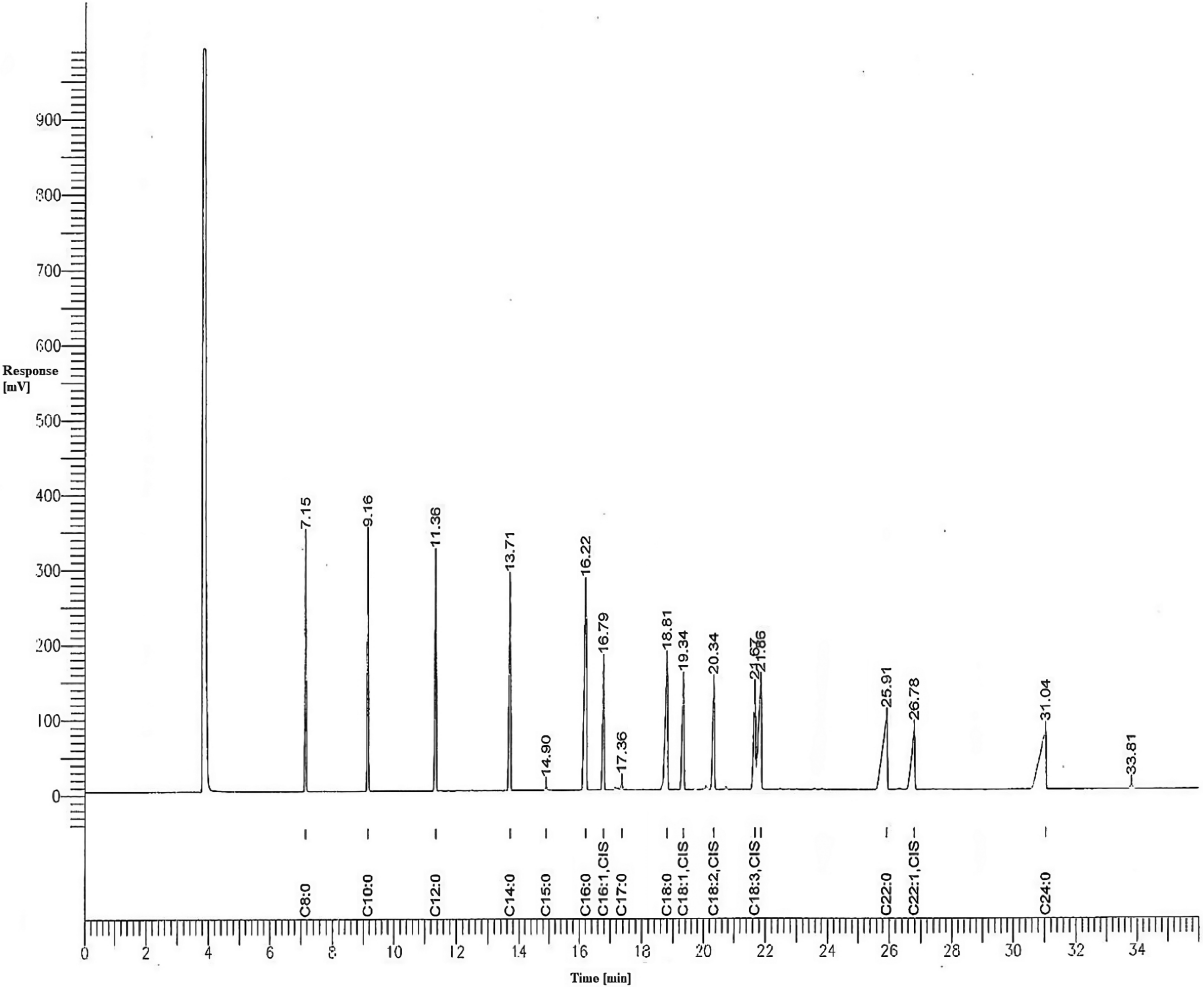
Fatty acid profile of FAME standard mixture w/w % (Sigma‐Aldrich Company Ltd., Lipid Standard, FAME‐189‐18).

**FIGURE 2:**
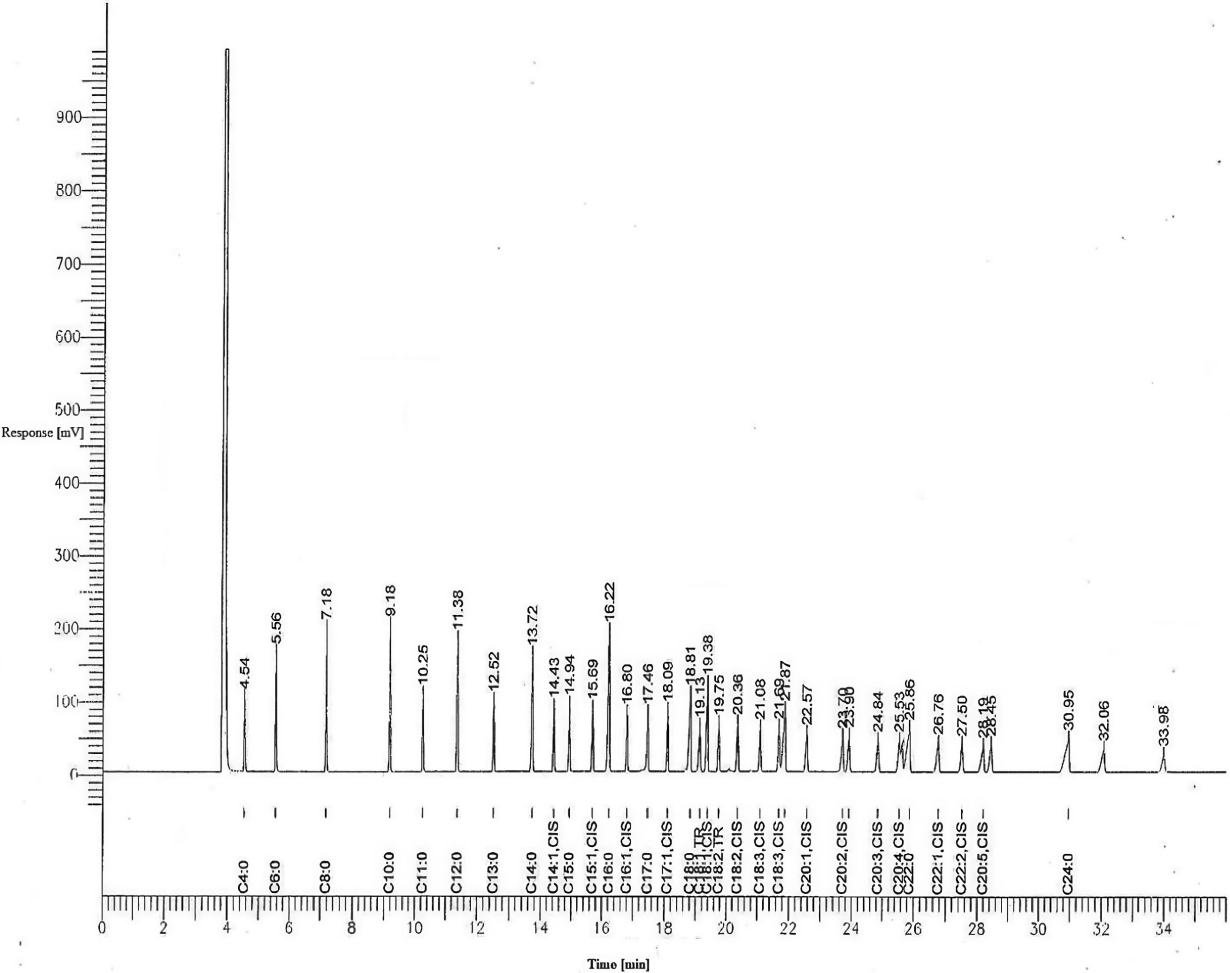
Fatty acid profile of the FAME standard mixture w/w% (Sigma‐Aldrich Company Ltd., Lipid Standard, FAME‐189‐19).

Moisture, lactose, fat, and protein contents of the lyophilized raw and pasteurized camel and cow milk samples (*n =* 6) were determined using the AOAC standard methods. Vitamin‐C and fat in raw and pasteurized camel milk samples (*n* = 6) were extracted following the earlier method of Zibaee et al. (2015). Fatty acid methyl esters (FAMEs) were separated using gas‐liquid chromatography by using a BPX70 SGE FAME capillary column (length, 50 m × 0.22 mm ID) with 70% cyanopropyl polysiloxane (equivalent). The carrier gas (N2) inlet pressure was 12.0 psi (75 KPa), injector mode was splitless, at 240–270°C temperature in the injector and at 280–300°C in the FID detector with 0.5 μL injection sample size. A ramped temperature was programmed: the initial temperature was held at 80°C for 3–5 s and increased from 160, 210 to 220°C at 10, 5, and 1°C/min held for 3–5 s, then heated and held for 5 and 3 min, respectively. The separated FAMEs were identified against FAME standards (Sigma‐Aldrich Company Ltd.; Figures 1 and 2).

Some saturated fatty acids (SFAs) are associated with coronary heart disease risk. This risk can be evaluated using the atherogenicity index, as described in the review by Konuspayeva et al. (2008). The index of atherogenicity (IA) was calculated using the following equation:

$$\mathrm{IA}={\text{34𝑎34𝑎𝑆}}_{12}+{\mathrm{bS}}_{14}+{\mathrm{cS}}_{16}/\mathrm{dP}+\mathrm{eM}+{\mathrm{fM}}^{\prime },$$

wherein S_12_ = C12:0, S_14_ = C14:0, and S_16_ = C16:0; *P* = sum of ω6 and ω3 polyunsaturated fatty acids (PUFAs); *M* = oleic acid; and *M*′ = sum of other monounsaturated fatty acids (MUFAs). Furthermore, *a*–*f* are empirical constants: *b* = 4 and *a*, *c*, *d*, *e*, and *f* equal to 1.

### 2.5 Statistical analyses

All data are expressed as mean ± SEM. The results from different variables were statistically analyzed by two‐factor without replication ANOVA using MS Excel from Microsoft Office 365 (Microsoft Corporation). This comparison of different groups at *p* < 0.05 was considered statistically significant.

## 3 RESULTS

### 3.1 Effect of camel milk on body weight

**FIGURE 3:**
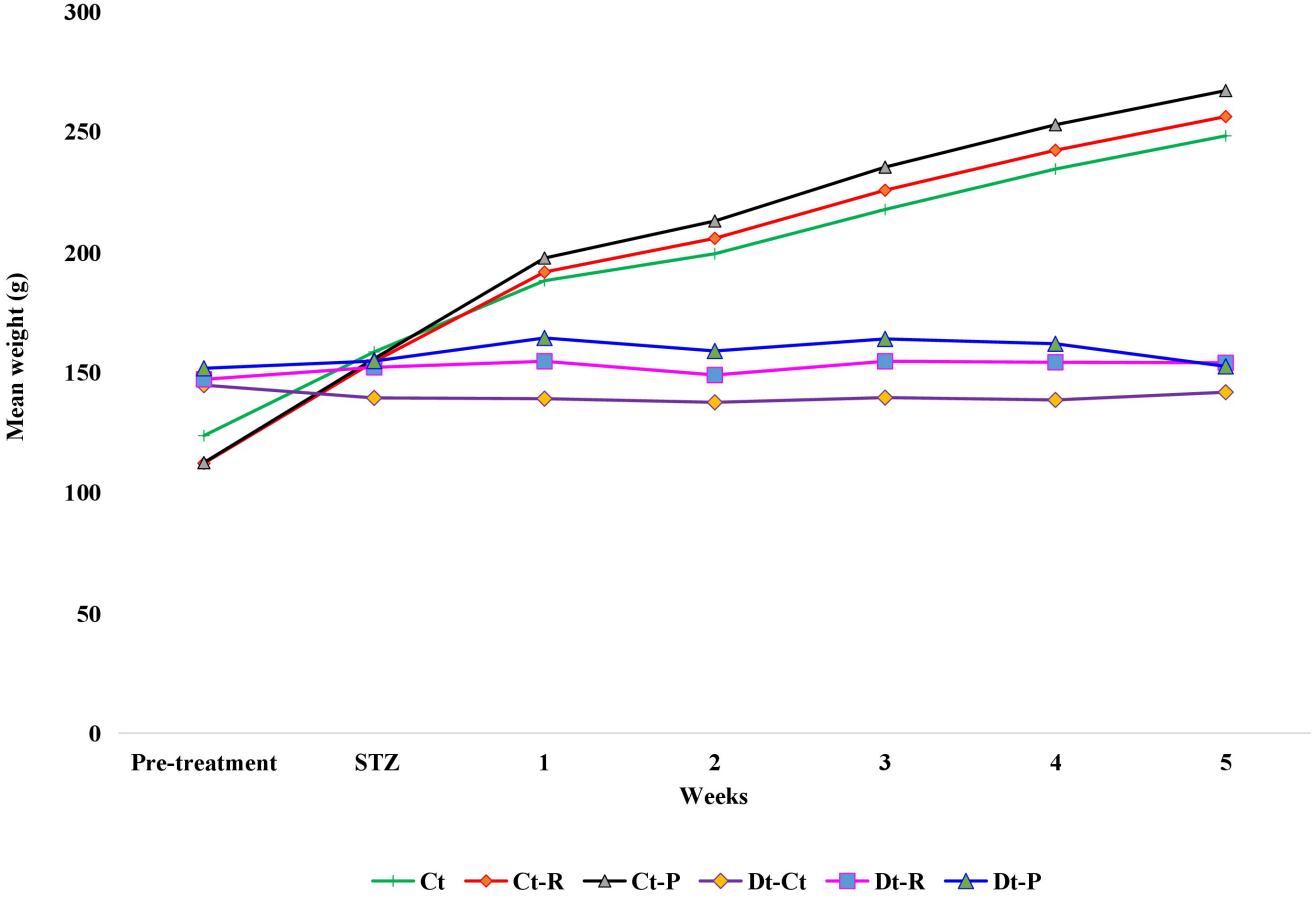
Weekly variations of weight in non‐diabetic and diabetic rats fed with raw and pasteurized camel milk. Nondiabetic control rats (Ct), nondiabetic control rats fed raw camel milk (Ct‐R), nondiabetic control rats fed pasteurized camel milk (Ct‐P), diabetic control rats (Dt‐Ct), diabetic rats fed raw camel milk rats (Dt‐R), diabetic rats fed pasteurized camel milk (Dt‐P). ***Mean significance at *p* < .001.

Feeding raw or pasteurized camel milk (150 mL/d) for 5 weeks revealed only negligible changes in the body weight of the Ct‐R and Ct‐P rats compared with that of the nondiabetic control rats (Ct). Interestingly, the diabetic rats fed with raw camel milk (Dt‐R) showed significant weight gain over the diabetic rats fed with pasteurized milk (Dt‐P) (*p* < .001). The preference for drinking the entire volume of raw milk over pasteurized milk attributed to such weight gain in diabetic rats. Comparatively, the weight of rats in the Dt‐Ct group was significantly lower than that of the non‐diabetic control rats (Ct) within the same period of the experiment (Figure 3). This was also validated statistically by ANOVA tests (Table 1).

**TABLE 1:** ANOVA tests on the rats' weight with control and diabetic rats fed with camel's milk.

Source of variation	SS	df	MS	*F*	*p*‐value	*F* crit
Ct:Dt:R:P	27190.46	5	5438.09	7.40	.000126	2.53
1–5: weeks treatment	23285.41	6	3880.9	5.28	.000806	2.42
Error	22020.11	30	734.003			
Total	72495.99	41				

Abbreviations: Ct, control rats; Dt, diabetic induced rats; P, pasteurized milk; R, raw milk.

### 3.2 Biochemical analysis

**FIGURE 4:**
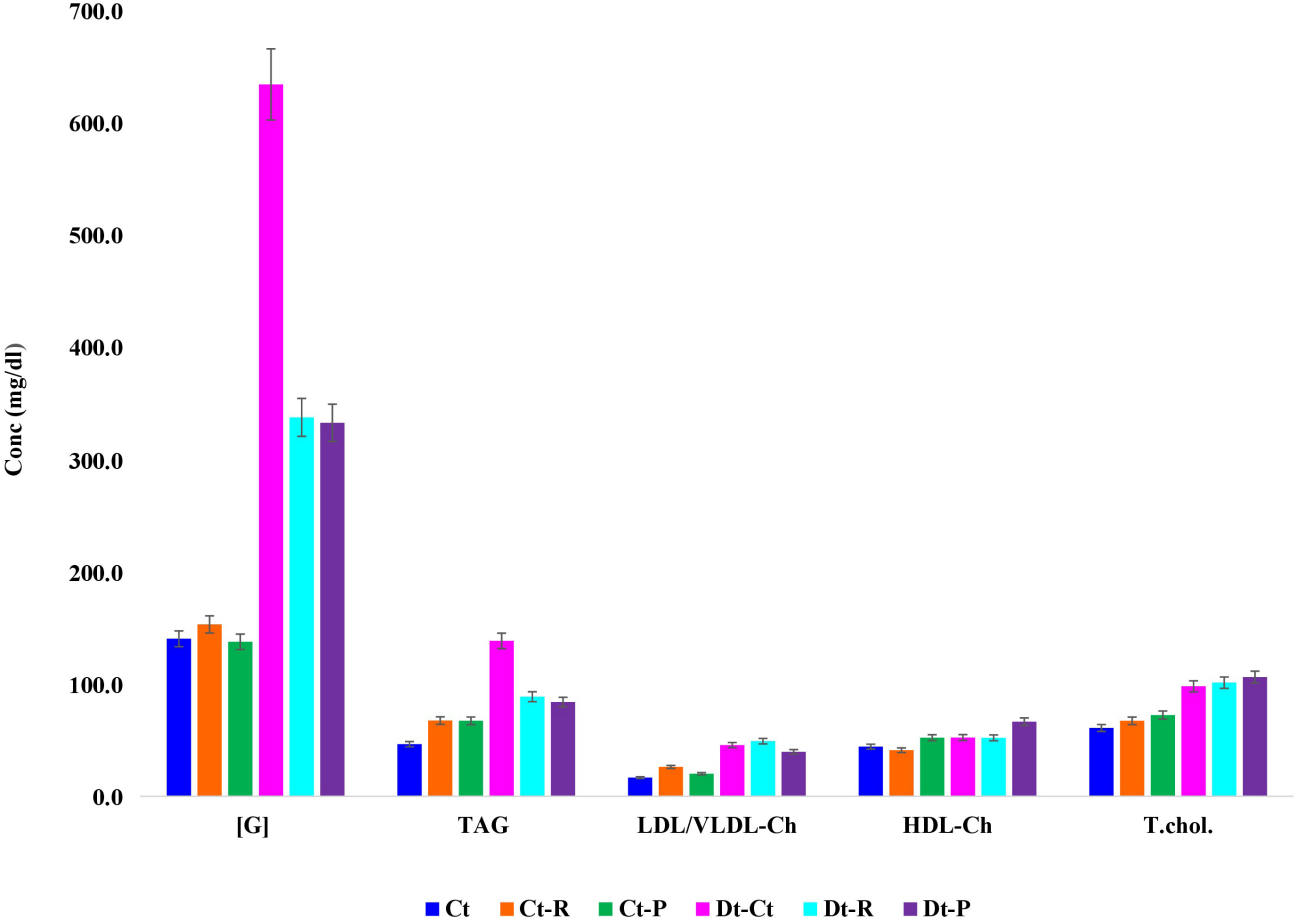
Variations of blood glucose, TAG, LDL/VLDL‐Ch, HDL‐Ch, and total cholesterol concentrations (mg/dL) in non‐diabetic and diabetic rats fed with raw and pasteurized camel milk. Nondiabetic control rats (Ct), nondiabetic control rats fed raw camel milk (Ct‐R); nondiabetic control rats fed pasteurized camel milk (Ct‐P), diabetic control rats (Dt‐Ct), diabetic rats fed raw camel milk (Dt‐R), and diabetic rats fed pasteurized camel milk (Dt‐P). ***Mean significance at *p* < .001.

The effect of raw and pasteurized camel milk on blood glucose, TAG, HDL‐Ch, LDL/VLDL‐Ch, and total cholesterol levels in rats was determined. Results for diabetic animals treated with raw (Dt‐R) and pasteurized camel milk (Dt‐P) were compared with those for non‐diabetic animals treated with raw (Ct‐R) and pasteurized camel milk (Ct‐P). These results were then compared with those of diabetic control (Dt‐Ct) and nondiabetic control rats (Ct). The serum glucose level was insignificant in nondiabetic (Ct‐R and Ct‐P) rats fed with raw or pasteurized camel milk for 5 weeks, and we attributed such results to the previous study (Hinz et al., 2012). In contrast, the serum glucose level of the Dt‐R or Dt‐P group was significantly (*p* < .001) lower (−47% and −48%, respectively) than that of the Dt‐Ct group, as noted in Figure 4. These results showed that both raw and pasteurized camel milk had the same hypoglycemic effect (*p* = .88), indicating that pasteurization did not alter the milk composition (Figure 4). Similarly, the serum TAG level increased nonsignificantly in the animals of the Ct‐R and Ct‐P groups, whereas it reduced significantly (*p* < .01) in the Dt‐R (−39%) and Dt‐P (−38%) groups, irrespective of raw and pasteurized camel milk fed to rats (Figure 4). In addition, both raw and pasteurized camel milk had a non‐significant effect on serum LDL/VLDL‐Ch levels of Ct‐R, Ct‐P, Dt‐R, and Dt‐P rats (shown in Figure 4) and no effect on blood HDL‐Ch level of Ct‐R and Ct‐P rats. However, pasteurized camel milk significantly (*p* = .05) increased (+49%) the serum HDL‐Ch level of Dt‐P rats. In contrast, the serum HDL‐Ch level of Dt‐R rats increased non‐significantly (*p* = .34) compared with that of D‐Ct rats (Figure 4). No significant difference was noted between the total cholesterol levels of the Ct and Ct‐R groups (*p* = .65). However, the total cholesterol level in Ct‐P rats was significantly (*p* < .05) higher (+10.5%) than that of Ct rats (Figure 4). These data indicated that neither raw nor pasteurized camel milk influenced the serum total cholesterol level of both Dt‐R and Dt‐P rats. ANOVA tests validated the significance of weight between the control, STZ‐treated, and raw and pasteurized camel milk‐supplemented rats (Table 2).

**TABLE 2:** ANOVA tests on raw and pasteurized camel and cow's milk.

Source of variation	SS	df	MS	*F*	*p*‐value	*F* crit
TGHLC	257738.62	4	64434.66	9.88	.0001	2.87
Ct:Dt:R:P	64730.66	5	12946.13	1.98	.12	2.71
Error	130474.99	20	6523.75			
Total	452944.27	29				

Abbreviations: C, cholesterol; Ct, control; Dt, diabetic; G, glucose; H, high‐density lipoprotein; L, low‐density lipoprotein; P, pasteurized milk; R, raw milk; T, triacylglycerol (TAG).

### 3.3 Comparative study of raw and pasteurized camel and cow milk

This study investigated how to compare and validate selected variables between camel and cow milk. Data showed that the method of pasteurization did not affect the nutrient composition of camel and cow milk (Table 3). The moisture, lactose, and vitamin‐C contents of raw and pasteurized camel milk were significantly higher than those of cow milk. The fat content in raw cow milk was significantly higher (*p* < .001) than that of raw camel milk, whereas the difference in fat content between pasteurized camel and cow milk was insignificant. No significant difference in protein content was noted between raw camel and raw cow milk, whereas the protein content of pasteurized cow milk was higher (*p* < 0.05) than that of pasteurized camel milk (Table 3). Statistical tests by ANOVA revealed a significant difference between the moisture, lactose, fat, protein, and vitamin‐C in raw and pasteurized camel as well as in cow's milk (Table 5a,e,i,m). However, no significance was observed between raw and pasteurized cow and camel milk for each variable (Table 5b,f,j,n,p).

**TABLE 3:** Composition of camel and cow milk.

Component	Camel milk		Cow milk	
	Raw	Pasteurized	Raw	Pasteurized *n* = 6
Moisture%	90.68 ± 0.78	89.60 ± 1.11	85.09 ± 0.97^a^	86.53 ± 0.71^b^
Lactose%	3.71 ± 0.06	3.11 ± 0.35	2.42 ± 0.05^a^	2.13 ± 0.16 ^b^
Fat%	3.42 ± 0.14	3.41 ± 0.11	4.93 ± 0.03^a^	4.17 ± 0.38
Protein%	3.22 ± 0.07	3.23 ± 0.09	3.40 ± 0.20	3.52 ± 0.07 ^b^
Vitamin C (mg/100 mL)	11.08 ± 0.25	11.06 ± 0.20	2.96 ± 0.37^a^	2.08 ± 0.11^c^

*Note*: Significant: ^a^ *p* <.05, ^b^ significant: *p* <0 .001, significant: ^c^ *p* <0.001, ±: mean standard error.

### 3.4 Comparative study of the fatty acid profile of raw and pasteurized camel milk

The percentage composition of each fatty acid except arachidonic acid (C20:4), the major component—palmitic acid—of SFAs, the percentage of total longchain oddnumbered carbon SFAs (C15:0, C17:0, and C21:0), the ratio of unsaturated fatty acids (USFAs) to SFAs, the total percentage of MUFAs, the percentage of oleic acid (C18:1 *cis* 9), the total percentage of PUFAs (C18:2, C18:3, C20:4, C22:5 and C22:6), the percentage and the ratio of ω‐6 PUFAs and ω‐3 PUFAs, and the IA analyzed in the raw and pasteurized camel milk are evidenced in the self‐explanatory Table 4. Statistical tests by ANOVA revealed a significant difference between the varied fatty acids in raw and pasteurized camel milk (Tables 5c,d).

**TABLE 4:** Fatty acid percentage of camel and cow milk (w/w %).

Fatty acid	Camel milk %		Cow milk % *n* = 6	
	Raw	Pasteurized	Raw	Pasteurized
C8:0	—	—	0.83 ± 0.09	BDL
C10:0	—	—	2.90 ± 0.43	1.38 ± 0.16^d^
C12:0	1.18 ± 0.08	1.16 ± 0.07	4.94 ± 0.23^b^	3.61 ± 0.23^d,g^
C14:0	10.79 ± 0.48	10.82 ± 0.52	12.11 ± 0.20^a^	11.00 ± 0.72
C14:1 *cis* 9	1.21 ± 0.33	1.23 ± 0.24	2.10 ± 0.11^a^	1.33 ± 0.10^e^
C15:0	1.43 ± 0.04	0.95 ± 0.34	1.75 ± 0.05^b^	1.52 ± 0.13
C15:1 *cis* 10	0.62 ± 0.02	0.61 ± 0.02	0.35 ± 0.00^b^	0.39 ± 0.03^g^
C16:0	26.75 ± 0.26	26.90 ± 0.71	32.24 ± 0.46^b^	32.41 ± 0.15^g^
C16:1 *cis* 9	9.28 ± 0.87	8.80 ± 0.80	2.90 ± 0.06^b^	2.27 ± 0.19^c,g^
C17:0	1.24 ± 0.16	1.27 ± 0.27	1.08 ± 0.02	1.06 ± 0.14
C17:1 *cis* 10	0.73 ± 0.04	0.74 ± 0.04	0.49 ± 0.02^b^	0.38 ± 0.01^d,g^
C18:0	11.09 ± 0.31	10.98 ± 0.39	10.55 ± 0.17^a^	9.59 ± 0.12
C18:1 *trans* 9	3.52 ± 0.45	BDL	4.50 ± 0.05	2.29 ± 0.1^e^
C18:1 *cis* 9	22.14 ± 1.20	21.47 ± 2.04	23.02 ± 1.41	21.02 ± 0.58
C18:2 *trans* 9,12	0.39 ± 0.04	0.41 ± 0.02	0.51 ± 0.10	0.47 ± 0.03
C18:2 *cis* 9,12	2.17 ± 0.04	2.11 ± 0.07	3.98 ± 0.06^b^	3.24 ± 0.23^c,f^
C18:3 *cis* 6,9,12	0.39 ± 0.03	0.41 ± 0.06	0.24 ± 0.03^a^	0.42 ± 0.1
C18:3 *cis* 9,12,15	0.35 ± 0.02	0.45 ± 0.09	0.46 ± 0.02^a^	0.44 ± 0.09
C20:1 *cis* 11	0.34 ± 0.02	0.33 ± 0.02	0.40 ± 0.01	0.27 ± 0.1
C21:0	3.71 ± 0.73	2.78 ± 0.53	0.37 ± 0.01^b^	BDL
C20:4 *cis* 5,8,11,14	0.24 ± 0.00	0.21 ± 0.01^a^	0.28 ± 0.01^b^	0.23 ± 0.01^e^
C22:6 *cis* 4,7,10,13,16,19	0.54 ± 0.06	0.60 ± 0.11	0.35 ± 0.05^a^	0.35 ± 0.04
Total SFA	56.19 ± 3.32	54.86 ± 3.39	65.81 ± 3.35	52.91 ± 4.00
Total USFA	41.92 ± 1.69	37.37 ± 1.79	37.63 ± 1.63	32.44 ± 1.68
MUFA	37.84 ± 3.35	33.18 ± 3.30	31.76 ± 2.64	27.04 ± 2.98
PUFA	4.08 ± 0.31	4.19 ± 0.32	5.87 ± 0.56	5.40 ± 0.45
ω‐3 PUFA	0.89 ± 0.10	1.05 ± 0.18	0.89 ± 0.07	0.81 ± 0.12
ω‐6 PUFA	2.80 ± 0.52	2.73 ± 0.07	4.51 ± 1.04	3.49 ± 0.84
USFA:SFA	0.75	0.68	0.57	0.61
ω6:ω3 PUFA	3:1	3:1	4:1	4:10
IA	1.87	1.93	2.62	2.41

*Note*: Significance to raw camel milk: ^a^ *p* < .05, ^b^ *p* < .001; significance to raw cow milk: ^c^ *p* < .05, ^d^ *p* < .01, ^e^ *p* < .001; significance to pasteurized camel milk: ^f^ *p* < .01, ^g^ *p* < .001, results are mean ± SEM.

Abbreviation: BDL, below detectable limits.

**TABLE 5:** ANOVA tests on raw and pasteurized camel and cow's milk.

Source of variation	SS	df	MS	*F*	*p*‐value	*F* crit
Camel raw vs camel pasteurized						
MLFPV	11611.40	4	2902.84	24470.80	.0015	6.38
b Cm R Vs Cm P	0.28	1	0.28	2.43	.19	7.70
Error	0.47	4	0.11			
Total	11612.16	9				
c FA + IA	11121.46	28	397.19	450.47	.00134	1.88
d Cm R Vs Cm P	4.54	1	4.54	5.15	.031	4.19
Error	24.68	28	0.88			
Total	11150.69	57				
Cow raw vs cow pasteurized						
e MLFPV	10925.15	4	2731.28	6248.79	.0017	6.38
f Cw R vs Cw P	0.01	1	0.01	0.03	.86	7.70
Error	1.74	4	0.43			
Total	10926.91	9				
g FA + IA	11121.62	28	397.20	118.19	.00155	1.88
h Cw R vs Cw P	22.84	1	22.84	6.79	0.014	4.19
Error	94.09	28	3.36			
Total	11238.56	57				
Camel raw vs cow raw						
i MLFPV	11173.37	4	2793.34	339.98	.0012	6.38
j Cm R vs Cw R	17.71	1	17.71	2.15	.21	7.70
Error	32.86	4	8.21			
Total	11223.95	9				
k FA + IA	12241.62	28	437.20	92.95	.0014	1.88
l Cm R vs Cw R	1.98	1	1.98	0.42	.522	4.19
Error	131.70	28	4.70			
Total	12375.31	57				
Camel pasteurized vs cow pasteurized						
m MLFPV	11301.04	4	2825.26	358.85	.002	6.38
n Cm P vs Cw P	14.35	1	14.35	1.82	.24	7.70
Error	31.49	4	7.87			
Total	11346.89	9				
o FA + IA	9907.03	28	353.82	121.54	.0015	1.88
p Cm P vs Cw P	1.53	1	1.53	0.52	.473	4.19
Error	81.51	28	2.91			
Total	9990.08	57				

Abbreviations: Cm, camel; Cw, cow; FA, fatty acids; IA, index of atherogenicity; MLFPV, moisture, lactose, fat, protein, Vitamin‐C; P, pasteurized milk; R, raw milk.

### 3.5 Analysis of fatty acid content in raw and pasteurized cow milk

Comparative analyses of SFAs in raw and pasteurized cow's milk revealed: (a) even‐numbered long‐chain SFAs (C12:0, C14:0, C16:0, and C18:0), (b) the percentage of odd‐numbered long‐chain SFAs (C15:0, C17:0, and C21:0), (c) the total percentage of SFAs, (d) the ratio of USFAs to SFAs, (e) the percentage of MUFAs, the percentage of ω‐6 PUFAs and ω‐3 PUFAs, the ratio of ω‐6:ω‐3, and the significant percentages of fatty acids (C10:0; C12:0; C14:1; C16:1; C17:1 *trans* 9; C18:2 *cis* 9,12; and C20:4 *cis* 5,8,11,14) evidenced besides their high IA percent in raw and pasteurized cow milk (Table 4). Statistical tests by ANOVA revealed a significant difference between raw and pasteurized cow's milk (Table 5g,h).

### 3.6 Analysis of fatty acid content in raw camel and raw cow milk

A comparison of the fatty acid content revealed that the percentage of C8:0 and C10:0 was 0.83 ± 0.09 and 2.9 ± 0.43, respectively, in raw cow milk, as opposed to the absence of these fatty acids in raw camel milk. Several fatty acids (C12:0; C14:0; C14:1; C15:0; C16:0; C18:1 *trans* 9; C18:2 *cis* 9,12; C18:3 *cis* 9,12,15; and C20:4 *cis* 5,8,11,14) were significantly (*p* < .001) higher in the raw cow milk than in the raw camel milk. Contrastingly, C15:1; C16:1; C17:1; C18:0; C18:3 *cis* 6,9,12; C21:0; and C22:6 *cis* 4,7,10,13,16,19 were significantly (*p* < .05 and 0.001) lower in raw cow milk than in raw camel milk. The total percentage of SFAs in raw camel milk (56.19%) was lower than that of raw cow milk (65.81%; Table 5). The total percentages of USFAs and their ratios (0.75) to SFAs in raw camel milk were higher than those ratios (0.57) in raw cow's milk (Table 4). The ratio of ω‐6 PUFAs to ω‐3 PUFAs in raw camel milk was 3:1, whereas in raw cow's milk, it was 4:1. The IA for raw camel milk was lower (1.87) than that of raw cow's milk (2.62). Statistical tests by ANOVA revealed the significance (3) between the varied fatty acids of camel and cow's raw milk (Table 5k). However, no significant difference was observed between the raw camel milk and the raw cow's milk (Table 5l).

### 3.7 Analysis of fatty acid content in pasteurized camel and pasteurized cow milk

Notably, C8:0 was absent in both pasteurized camel and cow milk. The percentage of C10:0 was 1.38% ± 0.16% in pasteurized cow milk, but it was absent in pasteurized camel milk. Several fatty acids (C12:0; C16:0; and C18:2 *cis* 9,12) were significantly (*p* < .05 and 0.001) higher in the pasteurized cow's milk than in the pasteurized camel milk. The fatty acids C15:1 *cis* 10, C16:1 *cis* 9, and C17:1 *cis* 10 were significantly (*p* < .001) lower in the pasteurized cow's milk than in the pasteurized camel milk. The ratio of USFAs to SFAs was 0.68 in pasteurized camel milk compared to 0.61 in pasteurized cow milk. The ratio of ω‐6 to ω‐3 in pasteurized camel and cow milk was 3:1 and 4:1, respectively. The IA of pasteurized camel milk (1.93) was lower than that of pasteurized cow milk (2.41) (Table 4). Statistical tests by ANOVA revealed the significance between the varied fatty acids in camel and cow's pasteurized milk (Table 5o). However, no significance was observed between the pasteurized camel milk and the pasteurized cow's milk (Table 5p).

## 4 DISCUSSION

STZ‐induced diabetic rats fed with raw (Dt‐R group) or pasteurized (Dt‐P group) camel milk for 5 weeks revealed no significant increase in their body weight (Figure 3, Table 1). This is in contrast to the earlier findings (Al‐Numair, 2010), implying that long‐term consumption of camel milk may be required for improving the body weight of diabetic rats.

This study showed that raw or pasteurized camel milk had an insignificant effect (Table 2) on the serum glucose, TAG, and LDL/VLDL‐Ch levels of nondiabetic rats (Ct‐R and Ct‐P groups) and was in line with the earlier findings of Al‐Numair et al. (2011). However, this study on STZ‐induced diabetic rats fed with raw (Dt‐R group) or pasteurized (Dt‐P group) camel milk for 5 weeks revealed an increase in HDL‐Ch and body weight (Figure 4, Table 2).

The serum glucose level of diabetic control rats (Dt‐Ct) was significantly (*p* < .001) higher (634.4 ± 43.61 mg/dL) than that of normal control (Ct) rats. The mean serum glucose level of the Ct group was 140.4 ± 10.73 mg/dL, consistent with that of earlier studies (Abdulrahman et al., 2016). The five‐week treatment of diabetic rats with raw (Dt‐R) and pasteurized (Dt‐P) camel milk showed a significant hypoglycemic effect (*p* < .001; 47% and 48%, respectively) compared with that of the control Dt‐Ct group (Figure 4). The insulin concentrations in camel milk is affected by the lactation stage, diet, and quantity of milk produced by camels (Abdulrahman et al., 2016; Ebaid et al., 2015). As camel milk does not coagulate in an acidic environment, insulin in the milk may be rapidly passed through the stomach into the small intestine, where it is more readily available for absorption (Shori, 2015; Wang et al., 2015) despite the marginal degradation of insulin by gut proteases. In addition, camel milk has been reported to contain insulin‐like growth factor‐1 (Wang et al., 2015) with many functional similarities with insulin. When this is administered to individuals with type‐1 DM, a reduction in insulin is observed (Ebaid et al., 2015; Wang et al., 2015).

This study showed that camel milk has a high vitamin‐C content (Table 3). Many studies have revealed that the daily consumption of vitamin‐C by diabetic patients may be instrumental in reducing blood glucose, total cholesterol, TAG, and glycosylated hemoglobin (Christie‐David et al., 2015). Vitamin‐C is a potential antioxidant and could help to fight oxidative bursts and decrease cardiovascular disease risk in diabetic individuals (Pehlivan, 2017; Singh et al., 2017). Further, a combination of insulin and vitamin‐C may impede blood vessel damage caused by diabetes. Camel milk contains high mineral content, including zinc, which mimics insulin action (Zibaee et al., 2015), causing an increase in insulin‐induced glucose transport (Abdulrahman et al., 2016; Mirmiran et al., 2017) and improving glycemic control. Zinc also promotes insulin action by exerting positive effects on insulin synthesis and secretion, and is required for the structural conformation of insulin (Mihic et al., 2016; Qaid et al., 2016). Feeding diabetic rats with raw (Dt‐R) or pasteurized (Dt‐P) camel milk significantly (*p* < .05) decreased serum TAG compared with those in D‐Ct rats (Figure 4). The reductions in serum TAG in the Dt‐R and Dt‐P groups were 36% and 39%, respectively. The deficiency of lipoprotein lipase (LPL) activity is known to contribute to the elevation of TAG in humans and animals with diabetes (Borén et al., 2020; Ejtahed et al., 2015; Park & Haenlein, 2021). Management of diabetes with insulin lowers plasma TAG by returning LPL activity to the normal level. Thus, the decrease in TAG following camel milk ingestion might be attributed to increased insulin secretion, which in turn increases LPL activity. Other factors such as high vitamin‐C content and zinc in camel milk may also lower blood TAG, total cholesterol, and LDL‐Ch (Al‐Numair et al., 2011; Ejtahed et al., 2015) and increase blood HDL‐Ch in patients with diabetes.

Raw or pasteurized camel milk did not significantly affect the serum HDL‐Ch level of non‐diabetic rats (Ct‐R and Ct‐P groups) compared with that in Ct rats. This was in agreement with the earlier findings (Al‐Numair, 2010). In contrast, an increase in HDL‐Ch was found in diabetic rats fed camel milk. This discrepancy may be because HDL‐Ch functions in both forward and reverse transport of cholesterol in rats. In this study, Dt‐R was insignificant, although a 16% increase in HDL‐Ch was observed. However, Dt‐P rats fed with pasteurized camel milk revealed an increase (47%) in the serum HDL‐Ch levels. In contrast to the earlier findings (Al‐Numair, 2010), this study suggested that raw or pasteurized camel milk had an insignificant effect on serum LDL/VLDL‐Ch in control non‐diabetic (Ct‐R and Ct‐P) or diabetic rats (Dt‐R and Dt‐P). This difference can be attributed to the different working techniques, rat strains, STZ doses, or experimental replicates. The treatment of rats with STZ is known to increase blood LDL‐Ch, VLDL‐Ch, and total cholesterol levels, which in part may be due to the hepatic recirculation of inflowing free fatty acids and other lipids. It could also be due to delayed disposal of VLDL‐Ch and chylomicrons and a decrease in the apoprotein moieties of lipoproteins necessary for triglyceride‐rich particles.

Raw camel milk had no effect on the total serum cholesterol level of control non‐diabetic rats (Ct‐R). However, pasteurized camel milk (Ct‐P) significantly (*p* < .05) increased (10.5%) the total serum cholesterol level. This increase may be attributed to an insignificant increase in serum HDL‐Ch in the Ct‐P group. Raw or pasteurized camel milk (Dt‐R and Dt‐P) did not change the total serum cholesterol levels in diabetic rats compared with that of diabetic control rats (D‐Ct). Thus, pasteurized camel milk reveals health‐beneficial effects and is recommended for nondiabetic subjects to maintain their cholesterol level. The effect of camel milk on the lipid profile could be partly attributed to the greater amount of stearic acid (C18:0) and MUFAs in camel milk than in cow milk (Kula, 2016). Stearic acid is known to significantly lower the total‐Ch, LDL‐Ch, and HDL‐Ch concentrations compared with those of other long‐chain SFAs. In addition, stearic acid and oleic acid (C18:1) revealed similar effects on serum lipoproteins (Amor et al., 2020). However, some studies have shown that the effect of stearic acid to lower HDL‐Ch is greater than that of other USFAs. Thus, stearic acid and oleic acid might have similar effects on LDL‐Ch and TAG, but may differ in their individual effect on HDL‐Ch. In addition, stearic acid does not change the total‐Ch/HDL‐Ch ratio, unlike carbohydrates. The effect of oleic acid and carbohydrates on total‐Ch concentration is similar, but oleic acid increases HDL‐Ch and lowers VLDL‐Ch and TAG concentrations, resulting in a significant decrease in the total‐Ch/HDL‐Ch ratio. Thus, because of the increase in HDL‐Ch and decrease in VLDL‐Ch concentration, MUFAs indicated a positive effect on atherosclerotic risk than carbohydrates. The IA of raw and pasteurized camel milk was lower than that of raw or pasteurized cow milk (Table 4). The IA is strongly associated with the onset of coronary heart diseases such as atherosclerosis. Atherogenic fatty acids include lauric (C12:0), myristic (C14:0), and palmitic (C16:0) acids. These fatty acids are lower in camel milk than in cow milk (Table 4). SFAs are thought to increase cardiovascular risk because they elevate serum total LDL‐Ch concentrations relative to MUFAs and PUFAs. The high fatty acids could also be the reason to cause mild to moderate sickness and allergies in some lactose‐sensitive children after the consumption of cow's milk (Pensabene et al., 2018; Polidori et al., 2021; Rasheed, 2017). Many well‐controlled studies (Athyros et al., 2018; Karaman et al., 2021; Malik et al., 2012) showed the relative of oleic acid—palmitic acid—increases the total and LDL‐Ch concentrations. Several studies (Agrawal et al., 2011; Hudspeth, 2018; Konuspayeva et al., 2008; Park & Haenlein, 2021) have concluded that myristic acid causes a greater increase in total cholesterol concentrations than oleic acid because it increases both LDL‐Ch and HDL‐Ch. Similarly, lauric acid also causes a greater elevation of total and LDL‐Ch concentrations than oleic acid but does not have any effect on HDL‐Ch and TAG concentrations. Considering the IA, camel milk contains an appreciable amount of PUFAs, and the ratio of ω6: ω3 was within the dietary recommendations of 2:1. This ratio explains the finding that raw and pasteurized camel milk significantly reduced serum TAG concentrations in diabetic rats, and yet pasteurized camel milk significantly increased serum HDL‐Ch. Clinical studies have shown that ω‐6 PUFAs decrease serum total‐Ch concentration (Agrawal et al., 2011). A replacement of 5% of the energy of SFAs by USFAs results in a 43% decrease in coronary heart disease (Hirano, 2018; Hudspeth, 2018). Omega‐3 PUFA have multiple beneficial effects on coronary heart disease risk: they increase plasma HDL‐Ch and lower TAG, LDL‐Ch, and VLDL‐Ch concentrations. The hypercholesterolemic effect of SFAs is attributed to an increased production or formation of VLDL‐Ch associated with higher activity of the hepatic enzyme Acyl‐CoA cholesterol acyl transferase and decreased plasma LDL‐Ch turnover associated with fewer LDL‐Ch receptors. Conversely, USFAs are found to increase LDL‐Ch receptors by one to three folds. These results are supported by both in vitro and animal studies.

Based on the above results, the second phase of the experiment was conducted to compare camel milk and cow's milk to validate the alternative use of camel milk over cow's milk for human consumption. However, this assessment on cow's milk was made without the experimental effect on Sprague–Dawley rats, since the indigenous cow's milk procured from the local outlets confounded to characterize their specific traits. Hence, comparisons between camel and cow's milk were made only with their biochemical analyses. The influence of the mild pasteurization process (63°C for 30 min) on the nutrient content was insignificant in both camel and cow's milk. The moisture contents of raw and pasteurized camel and cow milk obtained in this study were consistent with those reported earlier (Abdulrahman et al., 2016; Gader & Al‐Haider, 2016; Zibaee et al., 2015). The moisture content in camel milk increased to 91% under high temperature and water limitation conditions (Abrhaley & Leta, 2018). This attributes to the high moisture content in camel milk in the hot and dry climate of Kuwait. The lactose and protein content of raw and pasteurized camel milks (Table 3) were found in line with earlier studies (3.81%–5.2% and 2.70%–3.35%, respectively). Contrastingly, the lactose content in raw and pasteurized cow milk (Table 3) obtained in this study was lower than in the earlier studies (3.9% and 4.7%). However, the fat content in the raw and pasteurized camel and cow's milk (Table 3) was found to be slightly high on the upper range, in line with the earlier studies (2.95%–3.6%), indicating fatty deposition in the animal body feed (Abdulrahman et al., 2016).

Interestingly, this study showed no impact of pasteurization on the vitamin‐C level of either camel or cow milk. This result was consistent with the earlier research of Abdulrahman et al. (2016), who demonstrated that vitamin‐C concentrations were unaffected at high temperatures (72°C). The high vitamin‐C content obtained in this study could be attributed to factors such as lactation stage and parity (multiparous) camels that have higher concentrations of vitamin‐C in their milk than primiparous cows (Christie‐David et al., 2015). The vitamin‐C content in camel milk was found to be 4–6 times higher than in cow milk and, hence, camel milk is suggested for consumption in light of its health benefits, especially in arid areas where camel milk is available in abundance and where rich dietary sources of vitamin‐C are scarce. Statistical tests by ANOVA validate the significant and insignificant differences between the nutrient contents of raw and pasteurized camel and cow's milk (Table 5).

Dietary fatty acids play a key role in the various pathological conditions associated with DM, such as insulin resistance and atherothrombogenic risk (Konuspayeva et al., 2008). Both DM and dietary fats play a significant role in the development of dyslipidemia and atherosclerosis, besides ameliorating inflammatory responses by modulating the serum lipid profile (Zhu et al., 2016) and inducing free radical generation. Excess consumption of atherogenic SFAs under DM conditions can accelerate the atherosclerotic process and is thought to be harmful (Hirano, 2018).

Fatty acids in cow's milk were found to be less heat‐stable than those in camel milk as against the standard fatty acid profiles (Figures 1 and 2). Pasteurization of cow's milk significantly lowered some fatty acids (C10:0; C12:0; C14:1; C16:1; C17:1; C18:1 trans 9; C18:2 cis 9,12; and 20:4 cis 5,8,11,14) as opposed to camel milk, except arachidonic acid (C20:4), which significantly reduced after the pasteurization process. Short‐chain fatty acids (C8:0 and C10:0) were detected in raw cow milk, whereas C8:0 was not detected in pasteurized cow milk. This might be due to the low volatility of C8 fatty acid. Conversely, these short‐chain fatty acids were not detected in both raw and pasteurized camel milk. Even‐numbered long‐chain SFAs (C12:0, C14:0, and C16:0) were significantly higher in raw cow milk than in raw camel milk. In pasteurized cow milk, C12:0 and C16:0 fatty acids were significantly higher than those in pasteurized camel milk. Raw and pasteurized cow milk contained significantly higher amounts of palmitic acid than raw and pasteurized camel milk (Table 4). Comparatively, pasteurized milk is found to have lower fatty acids than raw milk because of the technological treatment that involves heat treatment, homogenization, fermentation, and storage (Paszczyk & Tońska, 2022). The ratios of USFAs to SFAs and the MUFA content in both raw and pasteurized camel milk were higher than those in raw and pasteurized cow milk. In contrast, the PUFA content in raw and pasteurized cow milk was higher than that in raw and pasteurized camel milk. The optimal ratio of dietary ω‐6 to ω‐3 has been recommended to be 2:1 to 3:1 (Gallagher, 2008). Therefore, camel milk provides a balanced content of ω‐6 and ω‐3 fatty acids. This was validated by ANOVA to determine the significant and insignificant differences between the fatty acids in raw and pasteurized camel and cow's milk (Table 5).

## 5 CONCLUSION

The outcomes of this study revealed the effectiveness of hypoglycemic and hypotriglyceridemic properties in camel milk that can be utilized as an alternative dietary substitute for cow's milk to ailing diabetic patients. Furthermore, fatty acid profiles suggest beneficial properties of consuming pasteurized camel milk, and calculated IA recommend camel milk as a better alternative to other milk.

## AUTHOR CONTRIBUTIONS

**Amal M. AL‐Saffar:** Conceptualization (lead); data curation (equal); formal analysis (equal); funding acquisition (lead); investigation (equal); methodology (supporting); project administration (lead); supervision (lead); visualization (equal); writing – original draft (lead); writing – review and editing (equal). **Nadia Y. Al‐Qattan:** Formal analysis (lead); investigation (equal); methodology (equal); visualization (equal). **Mona A. Al‐Sughayer:** Data curation (supporting); formal analysis (supporting); investigation (supporting); methodology (supporting); supervision (supporting); writing – original draft (supporting); writing – review and editing (supporting).

## FUNDING INFORMATION

This research was funded by the College of Graduate Studies, Kuwait University (YS/08).

## CONFLICT OF INTEREST STATEMENT

The authors of this MS declare no sources of conflict of interest.

## ETHICS STATEMENT

All study methods were carried out in accordance with relevant guidelines and regulations.

## PATIENT CONSENT

This study did not involve case studies with patients and hence, not applicable.

## Data Availability

The data that support the findings of this study are available on request from the corresponding author. A privacy and ethical contract with farm owners who supplied camel milk restricts the data being not public.
